# Enabling Food Environment in Kindergartens and Schools in Iran for Promoting Healthy Diet: Is It on the Right Track?

**DOI:** 10.3390/ijerph18084114

**Published:** 2021-04-13

**Authors:** Nasrin Omidvar, Mina Babashahi, Zahra Abdollahi, Ayoub Al-Jawaldeh

**Affiliations:** 1 Department of Community Nutrition, National Nutrition and Food Technology Research Institute (WHO Collaborating Center) and Faculty of Nutrition Sciences and Food Technology, Shahid Beheshti University of Medical Sciences, Tehran 1981619573, Iran; m.babashahi21@gmail.com; 2 Department of Nutrition, Ministry of Health and Medical Education, Tehran 1467664961, Iran; abdollahi_z@yahoo.com; 3 World Health Organization Regional Office for the Eastern Mediterranean, World Health Organization, Cairo 11371, Egypt; aljawaldeha@who.int

**Keywords:** food environment, children, policy, school, kindergarten, Iran

## Abstract

Enabling policies related to kindergarten and school food environments can be effective approaches in preventing childhood obesity. This study investigated policies and/or programs with direct or indirect effects on the food environment in kindergartens and schools in Iran. In this scoping review, we systematically searched PubMed, Scopus, and Web of science, as well as Iranian scientific search engines, including Scientific Information Database and Magiran from January 1990 to October 2020, to identify literature on policies/programs affecting the food environment in kindergartens and schools in Iran. A total of 30 studies and policy documents were included in this review relevant to eight policies/programs. These programs have helped to control food availability in schools, increase nutritional awareness, positively influence physical function and school performances, and reduce malnutrition in rural kindergartens. However, improving the food environment in schools and kindergartens requires proper revisions and local adaptation of many of these policies, strengthening of cross-sectoral collaborations, provision of necessary financial and human resources, and ensuring regular monitoring and evaluation. Reflecting on Iran’s health policies and interventions provides insight into the progress achieved and challenges faced. Lessons can benefit the country itself, as well as other countries with similar contexts.

## 1. Introduction

Childhood is a critical period to establish lifelong eating habits that influence future risk of diet-related non-communicable diseases (NCDs) [1]. Rapid semi-linear growth in children makes them more sensitive and vulnerable to undernutrition, hidden hunger, and excess weight, known as the triple burden of malnutrition [2,3]. In 2018, about 200 million under-five-year-old children suffered from wasting or stunting, at least 340 million had hidden hunger, and 18% of 5–19-year-old children and adolescents in the world were overweight and obese [4,5]. It is estimated that by 2025, the prevalence of overweight in preschool children will increase to 11% worldwide [6,7]. The pediatric population in Iran, like many other developing countries, suffers from the triple burden of malnutrition, which can endanger their survival, growth, and development. National surveys revealed that 4.6%, 4.3%, and 4.3% of children under five years of age are stunted, underweight, and wasted, respectively, and over 20% of school students have excess weight [8,9]. Micronutrient deficiencies are also prevalent in this age category, e.g., zinc and vitamin D deficiencies present in 14% and 62% of 6-year-old children and 11% and 76% of adolescents, respectively [10].

Food environments have gained prominence by many policymakers in low- and middle-income countries (LMIC) as an entry point to combat food insecurity and malnutrition [11]. Food environments are defined as the interface where people interact with the food system to acquire and consume foods [3,12]. Based on the United Nations Children’s Fund (UNICEF), the framework for children and adolescents’ food environment is comprised of two domains: external and personal [13]. The external food environment consists of all the physical places where individuals go to purchase or consume food. It reflects exogenous dimensions related to food price, availability, marketing and advertisements, and product properties [13]. Personal food environments refer to the accessibility, affordability, convenience, and preferences of individuals. Moreover, according to Swinburn’s definition (2013), food environment refers to “collective physical, economic, policy and sociocultural surroundings, opportunities and conditions that influence people’s food and beverage choices and nutritional status” [14]. Over the past decades, major changes in the food environments have been driven by technological advances, food industry development, food and agricultural policies, and economic, social, and lifestyle changes [14]. More processed foods and fast foods are now readily available and accessible in multiple settings throughout the day in larger portion sizes and at relatively low prices [14].

Children often have little control over their food environment [15], and living in unhealthy food environments can easily encourage them to follow low nutritional value dietary patterns [16]. In addition, children’s food choices can be influenced by their sociocultural and economic context [17]. Studies in Brazil found a higher intake of ultra-processed foods such as cookies, pastries, and sweetbreads, as well as carbonated sugar-sweetened beverages among children with mothers with high educational levels [18] and among households with higher income [19]. On the other hand, it has been reported that children from poorer backgrounds were significantly more likely to consume foods excessively high in calories, such as chips, fries, candies, and chocolate, at least once a week [20]. Moreover, a study in Iran showed that students’ snack consumption can be affected by their peer’s behavior through both social acceptance and role-modeling [21]. It has been shown that adolescents usually increase fast food consumption with increasing age due to fitting in the peers’ norms, peer acceptance, and peer pressure [22,23] and results in a tendency to unhealthy eating behaviors [24]. A study on Iranian students has shown that unhealthy snacking behavior can be associated with high socioeconomic status due to limited parental control, improper social norms, and low nutritional knowledge of students [25].

For school-aged children and adolescents, food environments in or surrounding schools play a significant role in their food choices and consumption by the types of foods and beverages available and accessible [14,26]. Considering that almost all children obtain some years of schooling, health promotion efforts in schools could positively impact eating behaviors and future disease risk [27]. Global recommendations for the prevention of NCDs, including the Global Action Plan for NCDs [28] and the Rome Declaration on Nutrition and Framework for Action [29], call for heightened action on nutrition in school settings. The World Health Organization (WHO)’s Report of the commission on ending childhood obesity (2016) recommends establishing standards for a meal and use mechanisms to safeguard public health from conflicts of interest [30]. Such policies offer an opportunity to ensure that foods made available to students in schools meet dietary guidelines [31,32]. The effectiveness of proper food environment policies in kindergartens and schools in improving children’s diet and food choices have been reported, and such approaches are even proposed as the most cost-effective diet-related approaches to NCDs prevention [27,28,33,34].

Within the last decade, a number of nutrition policies and programs have been initiated in Iran with the aim of improving children’s dietary intake and nutritional status. To the best of our knowledge, no reviews of the food environment policies in kindergartens and schools in Iran have been performed so far. This gap limits our understanding of children s’ food environments in the country. This scoping review aims to provide an overview of the purpose, content, implementation process of the existing policies related to kindergartens and schools’ food environment in Iran and to evaluate their effectiveness.

## 2. Materials and Methods

### 2.1. Conceptual Framework

**Figure 1:**
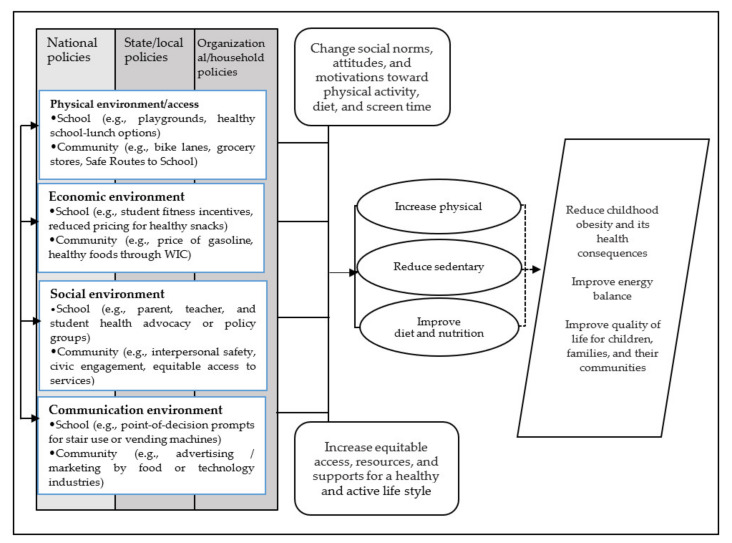
Conceptual framework (adapted from Brennan et al., 2011, with permission) [35]; WIC: Women, Infants, and Children program.

This review was guided by Brennan et al.’s conceptual framework (Figure 1), which demonstrates how the interaction of policies and environments influence children’s body weights, energy balance, physical activity, diet, and health outcomes [35]. This model considers four types of micro-environments, including physical (e.g., increase access to enhanced facilities and amenities), economic (e.g., reduced pricing for healthy snacks), social (e.g., campaigns, parent, teacher, and student health advocacy), and communication (e.g., increasing equitable access to resources and services; incorporation of existing or new social networks, promotional strategies, and positive media events, e.g., food advertising or marketing, festivals) [16,35].

### 2.2. Scoping Review Methodology

#### 2.2.1. Identifying the Research Question

The present scoping review aimed to answer the following research questions:

- What are the policies/programs implemented to improve the food environment of schools and kindergartens in Iran?
- What is the purpose of each of these policies/programs?
- According to the conceptual framework of the study, which dimensions of the food environment are covered by each of these policies/programs?
- What are the strengths, weaknesses, advantages, and limitations of each policy?
- What were the outputs or effectiveness of these policies?

#### 2.2.2. Identifying Relevant Studies

In this scoping review, we systematically searched articles related to policies related to the food environment in kindergartens and schools in Iran, using PubMed, Scopus, Web of science, and two Persian scientific search engines: Scientific Information Database (SID: www.sid.ir, accessed on 15 November 2020) and MagIran (www.magiran.com, accessed on 15 November 2020). Websites of the Ministry of Health and Medical Education (MoHME), the Ministry of Education (MoE), and the State Welfare Organization were also searched to identify additional information and data sources.

The literature search was adapted to the databases, and sources used and included the following subject heading terms and keywords: (Policy OR guideline OR regulation OR strategy OR law OR program OR plan OR intervention) AND (food environment OR food OR meal OR snack OR beverage OR drink OR diet OR nutrition) AND (kindergarten OR preschool OR school OR “primary school” OR “elementary school” OR “middle school” OR “high school”) AND (Iran). We limited the search to the following dates: 1st January 1990 to 31st October 2020 and applied no language restriction. Additional references were identified by searching the gray literature and hand searching the reference lists of the included articles.

#### 2.2.3. Study Selection

Inclusion criteria consisted of all articles that had information related to policies on the food environment of kindergartens and schools in Iran, e.g., the purpose(s), content, implementation process, strengths, weaknesses, advantages and limitations, and outputs or effectiveness. Clinical trials and studies that did not assess public health policies or conducted in settings other than kindergartens and/or schools were excluded. Screening of titles and abstracts was followed by full-text screening and data extraction.

#### 2.2.4. Charting the Data

Data extraction table was designed to enter and record the following variables: year of publication, publication type, name/title of policy/program, the type of food environment affected by the policy, target population, policy or intervention aims and objectives, description, results or outcomes (if applicable), weaknesses and limitations.

#### 2.2.5. Collating, Summarizing and Reporting Results

Included studies were reviewed, and information regarding the relevant policies was collected and summarized. In all stages, two of the authors (M.B. and N.O.) held regular meetings to discuss findings and reach a consensus about the management of findings.

## 3. Results

**Figure 2:**
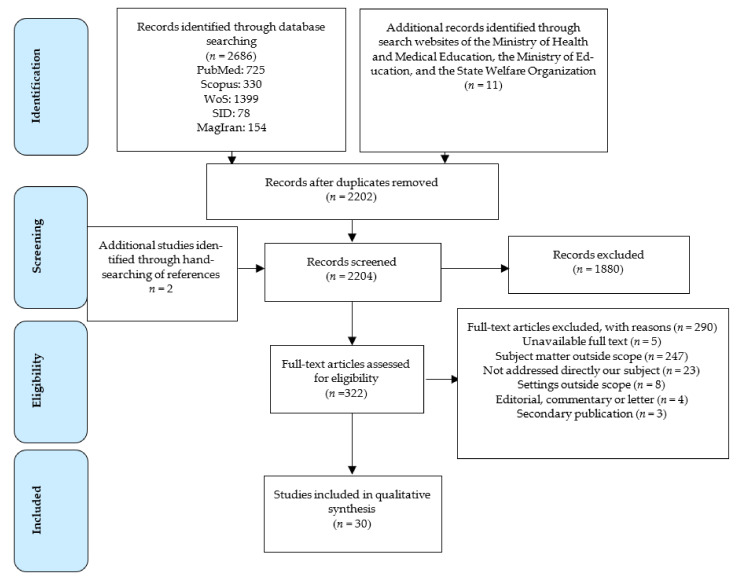
Flow diagram for the study selection process.

As illustrated in the PRISMA diagram (Figure 2), 2696 records were identified, 2202 records were screened, and 30 studies met the inclusion criteria and were included in the qualitative analysis. Overall, eight programs and policies that each affect kindergartens and schools’ food micro-environments were identified (Table 1). The following is a detailed description of these policies/programs.

### 3.1. Feeding Program—Providing One Warm Meal in Rural Kindergartens

In 2007, the Ministry of Welfare and Social Security, in collaboration with the MoHME and the Welfare Organization, formulated the provision of one warm meal in rural kindergartens as a strategy to improve the nutritional status of children aged 3–6 years of age in low-income families in rural areas. The program aimed at providing part of the targeted children daily nutritional needs, increasing their nutrition awareness as well as that of teachers and kindergarten managers, improving their healthy eating habits and behaviors, monitoring their growth through assessment of anthropometric indicators, and encouraging parents to send their children to kindergarten. In each province, the program is coordinated and supervised by the community nutrition office in the local medical universities. The meal’s dietary composition is adapted from the national desirable food basket (395 ± 50 kcal and 19.4 ± 4 g of protein) [37].

A quasi-experimental study evaluated the effects of the program on the growth of 1809 2–5-year-old children over a period of six months in rural areas of Birjand (the capital of South Khorasan province). The result of the study revealed that there was a 3% improvement in weight-for-height and 2% in weight-for-age indices of children after six months follow up [37]. However, the frequency of moderate stunting had increased, while there were small improvements in severe stunting (probably due to the short duration of the study). There was no significant change in the prevalence of obesity and overweight.

Despite the positive effects of the program on rural children’s growth status, it has failed to cover an important high-risk group of under-five children in the country, i.e., those living in poor suburbs [36]. Based on the executive instruction of this program, kindergartens in vulnerable areas in suburbs are covered if funding is provided.

### 3.2. The Ban Law of Food Marketing and Advertising in Kindergartens and Schools

The regulatory measures for restriction of marketing and advertisements of unhealthy food and sweetened beverages in kindergartens and schools approved by the Council of the Islamic Revolution Assembly in 1978 drew the limitations of these advertisements. According to article 12, no marketing and advertising are allowed in kindergartens and schools [38]. Therefore, according to this law, any food marketing in kindergartens and schools is banned. However, studies in different provinces, including Kerman and Tabriz, showed that in 35 and 9.7 percent of schools, respectively, there has been distribution of marketing brochures from supermarkets and fast food outlets and/or advertisement of candies, sweets, fast food, and soft drinks [42,61]. Moreover, a recent study in Tehran province showed that in 9.4 percent of schools, food products advertisements were posted on the facilities and equipment, e.g., refrigerator and/or freezer in the canteen environment that was provided to schools as promotion [39].

### 3.3. Healthy School Canteen Policy

Based on the available evidence regarding the high consumption of unhealthy and nutrient-poor foods by children [62,63] and availability of such foods in schools [64], in 2014, the national guidelines for healthy school canteen (HSC) by the MoHME and the Ministry of Education (MoE) was introduced [40]. The goals of this bylaw are increasing access to healthy snacks, preventing the supply of foods with low nutritional value, improving children’s nutrition patterns, providing a portion of required energy (300 kcal per day in snacks at school), protein and necessary nutrients, and maintaining and promoting health and prevention of NCDs [43]. In this bylaw, healthy food items are appropriate to one’s nutritional needs, are safe, varied, balanced, low in salt, low in fat, and have less than 5% of trans fatty acids. Based on the HSC guideline, all schools in Iran have to comply and provide healthy food and drink choices in their canteens [43]. In 2018, the revision of the HSC guideline ensured the promotion of fruits due to their low availability in schools’ canteens. However, serious educational work is needed to encourage students to consume fruit, and the high price of fruits is an obstacle that should be considered [39].

Two recent studies have provided evidence on the implementation of this bylaw. A cross-sectional study in Kerman province in central Iran showed low compliance of school canteens with HSC guidelines, with about half of food items in school canteens being unhealthy, e.g., chips, fried foods, and sugary drinks [25]. Another cross-sectional study in Tehran province in 2019 showed that after four years of implementing the HSC policy, more than 54% of available foods in 64 school canteens did not comply with the permitted food list of HSC guidelines. Cream biscuits, stuffed cookies, and coated cakes had the highest share among not permitted foods available and were sold in schools [39].

One of the main reasons behind the poor compliance of schools with this guideline is the fact that canteens are considered a source of income for some school principals [41]. A qualitative study using in-depth interviews with key informants of the HSC program showed that despite the improvements in the type of foods available in school canteens after initiation of this program, it has failed to be implemented completely. Based on the interviewees, this is mainly due to inadequate physical and economic infrastructure of schools to set up standard canteens, the high price of healthy foods and limited healthy alternatives, inadequate monitoring of school canteens, and conflict of interest between the actors [39].

### 3.4. National School Free Snack Program

The School Free Snack Program (NSFSP) in Iran dates back to the 1970s. The program was funded by the government and aimed to improve students’ nutritional status. Through this program, milk, nuts, and fruits were distributed for students free of charge [65]. After the 1979 revolution and several hiatus years, the program was resumed in the late 1990s with the collaboration of MoE, MoHME, and the Welfare Organization in primary schools of the deprived areas only [44]. The MoE is responsible for planning and providing food packages for the targeted schools, and the MoHME supervises students’ health and their health needs. In the meantime, the Welfare Organization and charities indirectly affect students’ nutritional status by providing financial support to their families. In each province, the activities of these organizations are coordinated and supervised by the provincial governors.

Based on the available evidence, the snacks provided through the NSFSP were nutritionally poor in terms of total energy and protein contents (provide 140 kcal and 2.5 g of protein/day) [44] and did not meet the minimum levels recommended as the standard snacks for school-age children (300–400 kcal/day and 5–10 g of protein/day) [66]. Moreover, students poorly consumed these snacks. This may have been associated with the lack of variety of distributed food items [44]. Currently, this program has been discontinued even in the deprived areas due to budget limitations.

### 3.5. School Milk Program

In 2001, the free school milk program was initiated for all schools nationwide under the National Milk Committee’s supervision. It aimed to promote milk consumption and improve students’ health and growth. The program was a collaboration between the MoE, MoHME, Planning and Budget Organization, Ministry of Industry, Mine and Trade, and National Standardization Organization [47].

Despite the positive effects of milk provision on students’ eating habits, physical growth, and school performance [36], there have been reports of poor acceptance and low compliance of students in more than one-third of high school students [45]. The most common reason for non-acceptance has been reported as the unfavorable taste of the milk distributed [45]. In addition, other factors, including poor quality, improper milk distribution schedule, and problems related to milk transportation and storage, are challenges that have negatively affected the implementation of the program [36,46]. Inadequate attention to the required resources and implementation of the program nationwide for all students, teachers, and school staff from preschool to high school are some of the most important factors that have hindered the school milk program continuity. Since 2018, due to funding problems and insufficient budget allocation, the school milk program has become limited to schools in the deprived areas [36].

### 3.6. Health-Promoting School (HPS) Program

The HPS program was launched in 2010 [25]. It was then quickly expanded from 72 schools in the pilot phase to about 20,000 within three years in all provinces [41]. This program comprises the provision of eight components within the school environment, including comprehensive health education, clinical services, healthy physical environment, physical activity, healthy nutrition (including both healthy school canteen and nutrition education), the school staff health promotion, mental health services and counseling, and participation of parents, students, school staff and community in health-promoting programs [9].

The results of studies examining the effectiveness of HPS in the food environment of Iranian schools have not been consistent [41,48,49,50,51,54]. Two studies, one in Urmia [48] and another in Babol [49], showed a significant increase in scores of healthy physical environment and nutrition improvement after implementing the program in schools. However, three studies in Kerman [25], Karaj [50], and Tehran [51] indicated that the HPS program’s establishment has not been effective in improving students’ nutrition and food intake. In the Kerman study, no significant difference was detected between the frequency of consumption of healthy and unhealthy snack items and snacking behaviors in HPS and non-HPS schools [41]. Easy accessibility of unhealthy snacks in HPSs similar to non-HSPs is a factor that probably affected their consumption [25]. In addition, poor infrastructure and limited equipment, e.g., lack of refrigerators in canteens and sub-standard physical environments, and limited human resources (i.e., nutrition/health officer) has made the full implementation of the program almost impossible [42,54]. Additionally, the quick expansion of the program might have damaged the monitoring and audit mechanism(s) [41]. Altogether, proper revisions in the HPS program may be necessary in order to improve its effectiveness of creating a healthy food environment and promoting sound dietary behaviors [51].

### 3.7. The IRAN-Ending Childhood Obesity (IRAN-ECHO) Program

IRAN-Ending Childhood Obesity (IRAN-ECHO) program was developed in 2016 within the framework of the WHO-ECHO recommendations [57] by considering life-course dimensions [59]. The program contains multicomponent interventions to improve population health and health equity in childhood obesity prevention [57] and is based on intersectoral collaboration among different organizations [59]. Increasing the number of schools with healthy canteens and kindergartens with a healthy eating plan are among the program’s objectives [57]. Holding nutrition education classes, development and distribution of educational materials for students, mothers, principals, and teachers in kindergartens and schools, monitoring of HSC and kindergarten canteens; limiting the supply of unhealthy snacks and fast foods by street food vendors around the schools; as well as anthropometric assessment of children in schools and kindergartens are some of the nutrition-related components of this program [57].

A study on 7149 students aged 7–18 years who participated in the pilot phase of the IRAN-ECHO program for six months in six cities using a validated questionnaire showed that the frequency of desirable nutrition-related knowledge after the intervention was statistically significantly higher compared to before the intervention (32.5% vs. 24.8%, *p* = 0.02) [58]. The mean score of attitude regarding obesity complications (73.09 vs. 74.78, *p*-value < 0.05) and practice in relation to low consumption of unhealthy snacks (46.39 vs. 48.02, *p*-value = 0.03) were statistically significantly increased after the intervention compared to baseline [58]. Evaluation of the implementation process showed that the coverage of school-age children in the provinces of Tehran, Isfahan, and Ardabil was 46%, 57%, and 52%, respectively [56]. Based on the qualitative assessment, the common obstacles of the implementation of the program from the stakeholders’ points of view were: weak intersectoral and intra-sectional cooperation, insufficient human resources and facilities, low economic status of students that make it difficult to follow a proper diet, and shortage of health-care providers in schools and barriers related to the providing psychological counseling [56]. The challenges of implementing this program from the parents’ points of view were their busy schedule associated with limited time to prepare healthy foods and manage their children’s activities and sleep time, lack of monitoring of healthy school nutrition, limited time of health staffs due to high workload for education and providing nutritional guidance, lack of psychological counseling and mental health interventions for overweight children and adolescents [56]. Since the program is in its early stages, more results are expected to be published in the coming years.

### 3.8. Weight and Obesity Control of Students (Kouch)

High prevalence of overweight and obesity in school-age children (about 21% according to CASPIAN-V, 2015 [67]) and concerns about subsequent health problems, i.e., NCDs in the community, has led the MoE in collaboration with the MoHME to develop this program. Kouch is a national program within schools that aims at increasing knowledge, improving attitudes and helping students manage their weight and have an active and healthy lifestyle. In this regard, promoting a healthy food environment in school and at home to control students’ calorie intake through nutrition education of students, parents, and teachers is emphasized. After the COVID-19 pandemic, these educational activities have been continued virtually through the Shad application designed by the MoE [68]. The program is a new opportunity to be used in enabling students and their parents for healthier food choices and dietary intake.

## 4. Discussion

This study reviewed national/subnational policies/programs that can affect the food environments of kindergartens and schools in Iran. Eight policies/programs were identified and reviewed, of which two had approached the food environment of both schools and kindergartens, five had directly addressed schools, and only one was specifically directed at rural kindergartens. There is a close relationship between some programs. For instance, the HPS program is an optional program that includes a proportion of schools throughout Iran. HPSs are usually the pioneer schools in implementing health programs, including the IRAN-ECHO program. In the meantime, the Kouch program follows IRAN-ECHO components in the school setting across the country. The target group of the IRAN-ECHO program includes not only students but also different groups in other settings of the community, e.g., school staff, mothers, and children in kindergartens. In other words, there are some intersections between several programs, and given the lack of resources, it may be better to prioritize between available programs and integrate some of them together. After all, despite the fact that the formation of healthy eating behaviors in the preschool years has a high potential to shape eating behaviors in later years and prevent obesity [69], there seems to be a gap with regards to kindergartens’ food environment policies in Iran.

Several programs and policies have focused on the school food environment through manipulating food availability (e.g., HSC), as well as provision of healthy foods (e.g., School Milk Program) and/or healthy snacks (NSFSP) in the country. In most cases, poor or incomplete implementation of the programs due to limited resources (financial, personnel) and poor infrastructure were the main barriers identified in their effectiveness. Analysis of the reviewed programs/policies revealed several elements for improvement. First, school-based programs in Iran are mostly implemented as a collaboration between the MoE and MoHME. While both ministries are quite important in ensuring a healthy school food environment, the involvement of other actors and stakeholders, specifically the department of agriculture, food industry, municipalities, and media, should be considered as an important factor to improve their effectiveness. In a review of the European policies related to the food environment in kindergartens and schools, Kovacs et al. have emphasized the importance of integrated parallel policies and the involvement of all relevant sectors and stakeholders in the success and effectiveness of these policies [70]. Long collaboration between the U.S Department of Agriculture (USDA) and the school meal program in the U.S. [71] is an example of the importance of the involvement of other sectors besides the health and education sectors in improving students’ dietary intake through increasing the availability of fruits, vegetables, and whole grains, and decreasing the total calories, sodium, and trans fat contents of the meals [72].

Secondly, based on the findings, even though some programs and policies have been implemented for a long time, still a large proportion of available foods in schools do not comply with authorized healthy food lists. There are similar experiences in many countries, e.g., India, the Philippines, Brazil, Mexico, Haiti, Guatemala, and South Africa, where foods sold or eaten in schools had low nutritional quality and included items high in sugar (cookies, sugary drinks, sweets), salt (chips, crackers), or fat (fried foods, ice cream, hamburgers, and pizza) [73,74,75,76,77]. Poor program monitoring, on the one hand, and high profitability and palatability of unhealthy foods, on the other hand, are considered the main culprits behind such findings [73,74,75,76,77]. In addition, in Iran, like many other LMICs, limited access to healthy food choices has been identified as a barrier to implementing many policies or interventions in school food environments [78]. A set of food policies and interventions are needed to help improve student food choice and prevent obesity [79]. Some examples of such policies and regulations that are better to run simultaneously include taxation of unhealthy foods and sugar-sweetened beverages, subsidies on healthy food, marketing restrictions on low-nutrient-density products typically sold to children, package redesign to eliminate cartoon characters and other appealing features on junk foods, menu labeling, school and community gardens/greenhouses, individual nutrition education, and point-of-purchase prompts for healthy eating [79].

Thirdly, one of the factors that affects students’ dietary patterns is the availability of unhealthy snacks in the area surrounding schools [70]. No evidence was found for food environment policies in the areas surrounding kindergartens and schools in Iran. A limited number of countries have considered this issue. For example, in Romania, there is restrictive legislation regarding the sale of food items within a radius of 100-meters of schools [70]. Previous studies have shown that the local food retails around schools may act as a compensatory alternative, may negatively influence children’s food choices, and have linked childhood obesity to fast food restaurant closeness to schools [80,81,82]. In addition, banning the sale of soft drinks at school could lead to an increased intake outside the school [70]. While the provision of healthy foods in schools may be the first priority, the adoption of laws to control the environment around schools can help to support children’s health and dietary intake.

Fourthly, while nutrition education has been defined as an essential component in most programs reviewed, it was often not implemented or partially implemented due to inadequately trained personnel, lack of health coaches in schools, and/or the high cost of supplying education aids, e.g., brochures, posters, and leaflets. The Food and Agriculture Organization (FAO) considers school-based food and nutrition education as efficient, supportive approaches for food environment policies if properly integrated within their strategies [83]. However, if nutrition education programs are not integrated into the school curriculums, fidelity to their implementation can be dependent on the interest, motivation, and time of school staff or teachers, and they may be interrupted when school food environment policies are weakly monitored [83]. In addition to integration, attention needs to be given to the content and coherence of the educational messages communicated across schools [68]. On the bright side, the launch of a hot-line system and using on-line applications such as “Shad” application with the Covid-19 pandemic identified a new channel for the provision of health and nutrition counseling and education for children, parents, and school staff in Iran [68].

Fifthly, the centralized and top-down approach of the existing health programs in Iran can be considered a good opportunity in formulating and implementing national standards and public policies to promote a healthy food environment [70]; however, it can hinder the possibility of local modification(s) of food environment policies based on local food preferences and availability. In the case of HPS, the top-down approach led to the resistance of schools to accept the program. Using this approach in the formulation and implementation of such policies may negatively affect the ownership and accountability of stakeholders [84].

Sixthly, program coverage and population selection affect interventions’ effectiveness in reaching desired outcomes. Considering the fact that not everyone is affected by nutritional problems, targeting the whole community will be highly costly and can be ineffective [85]; thus, well-defined target groups are critical for the sustainability, efficiency, and cost-effectiveness of community nutrition programs, particularly in conditions with limited resources [85]. The degree to which programs such as the NSFSP can be effective in providing part of the nutritional needs of malnourished or food-insecure children across the country is dependent on how properly it has targeted those in need. In many high-income countries, e.g., Finland, Sweden, and the U.S., providing free meals for all children in schools has had a direct and tremendous effect on children’s diet [70]. However, universal coverage will require a large budget that may not be realistic in LMICs. Therefore, to protect children’s right to a healthy food environment, as recommended by the UNICEF [86], authorities and power owners should provide more support for food environment policies to properly select populations with the highest need and who would benefit the most from the intervention.

Finally, most reviewed programs in Iran lack proper monitoring and evaluation plans. Several studies in other countries have also reported that school food policies were often not evaluated, or their impact was rarely assessed [70,87,88]. Existing policy evaluations have mainly considered school-level changes and did not assess the program’s impact on individual dietary intake [70]. The absence of evaluation and comprehensive monitoring mechanism deprive policymakers of access to objective evidence for making decisions on required changes and good practices.

Overall, despite the popularity of the food environment-related policies in kindergartens and schools around the world, in many cases, they are poorly implemented [89]. Policy implementation barriers can even be more complicated when students’ food preferences, limited resources, and a shortage of coordination, support, and communication are also taken into account. As a result, the implementation of recommended policies for healthy food provision in schools and kindergartens has been inconsistent and inadequate worldwide [90]. One important issue in the evaluation of these policies is the ambiguity of healthy/unhealthy food classification. The definitions and development of robust scientific criteria are required to determine permitted/not permitted foods and beverages provided or sold in kindergartens and schools. In this regard, the use of international or regional tools, e.g., the nutrient profile model of the WHO Regional Office for Eastern Mediterranean [91], can support proper classification. Governments in many countries, including Australia, Canada, India, Singapore, and New Zealand, have already started applying nutrient profile models for evaluating the nutritional quality of foods in school food standards [92].

Some general limitations related to the current policies included constrained use of the evidence-based approach, insufficient physical and economic infrastructure, inadequate monitoring, shortage of human resources, lack of policies’ evaluation, conflict of interest among actors due to financial profitability of unhealthy food selling, the high price of healthy foods, and shortage of healthy food alternatives.

To the best of our knowledge, this study is the first review that reports the enabling food environment policies and programs in kindergartens and schools in Iran. The findings can help policymakers and decision-makers to develop evidence-based policies to improve kindergartens and schools’ food environment. Our scoping review also has several limitations. The quality of the literature analyzed was not assessed in this study. Thus, this review’s conclusions are based on the existence of studies rather than their intrinsic quality. Moreover, publication bias (whereby some results of food environment assessment in kindergartens and schools are not to be published as yet) has to be taken into account.

## 5. Conclusions

Improving the food environment in educational settings requires multisectoral policies and regulations that meet children’s needs in the societal context with respect to their physical, psychological, and psychosocial development. Therefore, policymakers will require to involve all relevant stakeholders before implementing such intersectoral policies and seriously prevent any different interpretations of the programs.

The availability of enjoyable, safe, and nutritious food, supported by a healthy food environment, is fundamental to foster better diets in kindergartens and schools in Iran. Implementing standards and policies, together with developing the capacities of school communities, is a top priority in promoting healthy school food environments and meals around the country. A healthy food environment, together with food and nutrition education, fosters and supports better food choices and practices among the school community.

## Figures and Tables

**Table 1:** Characteristics of food environment policies in kindergartens and schools in Iran.

Title of the Program/Rule	Food Environment Affected by the Policy	Setting	Target Group (Years Old)	Year of Implementation	Aims and Objectives	Key Actors	Results or Outcome Measures	Weaknesses of the Program/Intervention	References
Feeding program—providing one warm meal in rural kindergartens	Physical and economic	Kindergartens	Children in rural kindergartens (2–5)	2007	Provide part of the nutritional needs of children Increase children’s nutrition awareness Improving healthy eating habits and behaviors in children Monitoring the growth of children and determining anthropometric indicators Enhance parents’ awareness about children’s nutrition and development Persuade and encourage parents to send their children to kindergarten Raising the awareness of kindergarten managers and coaches about children development and nutrition	Ministry of Welfare and Social Security, MoHME and Welfare Organization	A quasi-experimental study in Birjand: 3% improvement in weight-for-height and 2% improvement in weight-for-age indices of children	Lack of coverage of many deprived areas Did not include a control group Did not collect data on confounding factors such as food security, crowding index, household income	[36,37]
The ban law of food marketing and advertising in kindergartens and schools	Communication	Kindergartens and Schools	All children (2–18)	1978	The ban on all marketing and advertising in kindergartens and schools	Ministry of Culture and Islamic Guidance and MoE			[38]
Healthy school canteen policy	Physical	Schools	All students (7–18)	2014	Increase access to healthy snacks Prevent the supply of foods with low nutritional value Provide a portion of required energy (300 kcal per day in snacks at school), protein, and necessary nutrients	MoHME and MoE	In the Kerman study [25]: low compliance of school canteens with HSC guidelines, with about half of food items in school canteens being unhealthy, e.g., chips, fried foods, and sugary drinks In the study in Tehran: More than 54% of available foods in 64 school canteens did not comply with the permitted food list of HSC guideline [39]	Restricted fiscal resources of the schools and financial profitability of canteens	[25,39,40,41,42,43]
National School Free Snack Program	Physical and Economic	Schools	Students in deprived areas	Late 1990s	Improving the nutritional status of students	Welfare Organization/ MoHME/ MoE		Low nutrition value of distributed snacks Low variety of snacks	[44]
School milk program	Physical and Economic	Schools	All students (4–18)	2001	Promoting milk consumption Improving health level in students	MoE/ National Milk Committee/ MoHME/ Program and Budget Organization/ Ministry of Industry, Mine and Trade/ National Standardization Organization	A cross-sectional study in Yazd city on 703 students [45]: 37% of the students did not consume milk at all No correlation between the knowledge and acceptance of milk A correlation between the attitude and acceptance of milk 63% of students who had consumed milk noted benefits of milk for their health as the reason for its consumption. 64% of the students who had not consumed milk did not drink it for its bad taste.	Shortage of funds Low quality distributed milk in some cases Unfavorable taste Improper milk distribution schedule Problems related to milk transportation and storage	[36,45,46,47]
Health-promoting school program	Social, Communication and Physical	Schools	All students (7–18)/ school principals and staff/ parents	2011	Foster the healthy development of the whole school community Provide a framework for developing health promotion initiatives in a way that supports and enhances the implementation of the curriculum Support the planning, implementation, and evaluation of health-related activities under the school development planning process Enhance the links between schools and their communities	MoE /MoHME	Urmia [48] and Babol [49] studies: A significant increase in scores of healthy physical environment and nutrition improvement after implementing the program in schools (*p* < 0.05). Karaj [50] and Tehran [51] studies: Not effective in improving students’ nutrition and food intake (*p* > 0.05). The Kerman study [41]: No significant difference between the frequency of consumption of healthy and unhealthy snack items and snacking behaviors in HPS and non-HPS schools (*p* > 0.05)	Weak collaborations among responsible organizations lack of strong legal support Insufficiency of funding Ambiguity of rules about assessment and monitoring of students and school staff’s health The scarcity of health training programs for students’ parents or school personnel Shortage of human resources Inconsistency between the findings of the studies	[9,41,42,48,49,50,51,52,53,54,55]
The IRAN-Ending Childhood Obesity program	Social, Communication and Physical	Kindergartens and Schools	All children (2–18)	2016	Reduction in the incidence of childhood obesity and identification and treatment of preexisting obesity in children and adolescents	MoE /MoHME	The coverage of school-age children [56]: Tehran (46%), Isfahan (57%) and Ardabil (52%) The results of after intervention in the pilot phase (7149 students aged 7–18 years who participated for six months in six cities) [57]: The frequency of desirable nutrition-related knowledge significantly increased (32.5% vs. 24.8%, *p* = 0.02) The mean score of attitude regarding obesity complications significantly increased (73.09 vs. 74.78, *p*-value < 0.05) The mean score of practice in relation to low consumption of unhealthy snacks significantly increased (46.39 vs. 48.02, *p*-value = 0.03)	Weak intersectoral and intra-sectional cooperation Insufficient human resources and facilities Low economic status of students Shortage of health-care providers in schools	[56,57,58,59]
Weight and obesity control in students (Kouch)	Social and Communication	Schools	All students (5–18)	2020	Screening obese and overweight students and determining the body mass index of all students in the country in two stages Advocacy and attracting collaboration of all stakeholders for better implementation of the plan Changing students’ behavior and lifestyle to an active and healthy lifestyle 3% reduction in the number of obese and overweight students	MoE /MoHME			[60]

MoHME: The Iranian Ministry of Health and Medical Education; MoE: The Ministry of Education.
